# OP72 Clinical And Economic Impact Of The Availability Of Innovative Therapies For Advanced Lung Cancer In Latin America

**DOI:** 10.1017/S0266462325101281

**Published:** 2025-12-29

**Authors:** Andrés F. Cardona, Natalia Sánchez, Liliana Gutiérrez-Babativa, Leonardo Rojas, Jairo Zuluaga, Stella Martínez, Lucia Viola, Carlos Carvajal, Juliana Bogoya, Laura Prieto-Pinto, Daniel Samacá, Antonio Robles, Joshua Kock, Claudi Martín, Luis Corrales, Luis E. Raez, Vladmir Cordeiro de Lima, Suraj Samtani, Oscar Arrieta

## Abstract

**Introduction:**

Over the past decade, there has been an increase in the development of innovative cancer treatments that have positively impacted upon mortality rates. However, the approval process at the local level remains lengthy. This study estimated the clinical and economic impact of the availability of innovative therapies for treating advanced lung cancer in men in five Latin American countries.

**Methods:**

Using public data, we analyzed the relationship between available innovative therapies and age-specific mortality rates for Argentina, Brazil, Chile, Colombia, and Mexico through a regression model. By comparing the number of therapies approved by the United States Food and Drug Administration (FDA) with those approved by each local regulatory agency, we estimated the avoidable deaths (ADs) that could have been averted if innovations had been available. We calculated years of life lost (YLLs) based on life expectancy, median age of death, and ADs. Productivity loss was estimated using each country’s retirement age and the annual gross domestic product (GDP) per capita in 2022.

**Results:**

Between 2006 and 2021, the FDA approved 24 innovative treatments for 30 indications. By 2021, only 60 to 77 percent of these therapies were available locally. Total ADs, YLLs, and PL were USD8,694, USD114,477, and USD439,179,876, respectively. The most significant productivity losses were found in Brazil and Argentina due to the large Brazilian population and Argentina’s high GDP per capita. Despite its low population, Chile’s high GDP per capita resulted in considerable productivity loss. Colombia and Mexico showed a notable clinical impact, suggesting the advantages of early approval. Although the availability of FDA-approved therapies has increased, local time lags have recently increased.

**Conclusions:**

Our results highlight the potential of improving regulatory processes to increase the availability of innovative therapies in five Latin American countries. Accelerating the introduction of these therapies for advanced lung cancer presents a major opportunity to enhance survival rates, instilling a sense of hope and optimism while avoiding substantial productivity losses.

